# Environmental suitability models predict population density, performance and body condition for microendemic salamanders

**DOI:** 10.1038/s41598-018-25704-1

**Published:** 2018-05-14

**Authors:** Enrico Lunghi, Raoul Manenti, Manuela Mulargia, Michael Veith, Claudia Corti, Gentile Francesco Ficetola

**Affiliations:** 1 Biogeographie, Universität Trier Fachbereich VI, Raum- und Umweltwissenschaften, Trier, Germany; 2 Museo di Storia Naturale dell’Università degli Studi di Firenze, Sezione di Zoologia “La Specola”, Firenze, Italy; 3 Natural Oasis, Prato, Italy; 4 Department of Environmental Science and Policy, Università degli Studi di Milano, Milano, Italy; 5 Speleo Club Nuoro, Nuoro, Italy; 6 University Grenoble Alpes, CNRS, Laboratoire d’Écologie Alpine (LECA), F-38000 Grenoble, France

## Abstract

Species can show strong variation of local abundance across their ranges. Recent analyses suggested that variation in abundance can be related to environmental suitability, as the highest abundances are often observed in populations living in the most suitable areas. However, there is limited information on the mechanisms through which variation in environmental suitability determines abundance. We analysed populations of the microendemic salamander *Hydromantes flavus*, and tested several hypotheses on potential relationships linking environmental suitability to population parameters. For multiple populations across the whole species range, we assessed suitability using species distribution models, and measured density, activity level, food intake and body condition index. In high-suitability sites, the density of salamanders was up to 30-times higher than in the least suitable ones. Variation in activity levels and population performance can explain such variation of abundance. In high-suitability sites, salamanders were active close to the surface, and showed a low frequency of empty stomachs. Furthermore, when taking into account seasonal variation, body condition was better in the most suitable sites. Our results show that the strong relationship between environmental suitability and population abundance can be mediated by the variation of parameters strongly linked to individual performance and fitness.

## Introduction

Organisms show a strong diversity of distribution patterns: some species are limited to very narrow ranges such as mountain tops or islets, while others are widespread throughout entire continents. Multiple factors, both biotic and abiotic, interact to shape species’ ranges. Generally, a species occurs in areas where abiotic conditions are suitable (i.e., positive intrinsic growth rate), where biotic interactions allow the persistence of viable populations (i.e., positive total growth rate), and where dispersal and colonization are possible^1,2^. In the last decade, the combination of these concepts with species distribution models has boosted our understanding of the processes affecting species distribution, allowing us to assess the factors determining the limits of species ranges, and to predict distributional changes in response to past and future environmental changes^3^. However, within the range of a species, there is often a huge heterogeneity of environmental features (both biotic and abiotic), and such variability can have profound effects on populations. In the areas with highly-suitable environment, it is expected that individuals have a better fitness^4–6^. In the last years, several studies have analysed relationships between the spatial variation of environmental suitability within species ranges, and key parameters of populations, such as fitness and demography^7,8^, with many studies assessing correlations between environmental suitability (derived from correlative ecological niche models) and population abundance (reviewed in^9^). A recent meta-analysis summarized the relationship between environmental suitability, derived from species distribution models (SDM), and the spatial variation of population abundance across multiple species of plants, invertebrates and vertebrates, and consistently found the highest abundances in the most suitable sites^9^ (but see also^10^). However, such relationship can diverge from linearity. First, the relationship between observed abundance and the environmental suitability determined by abiotic factors is assumed to be triangular. A low suitability generally corresponds to low abundances, while a high suitability can correspond to either low or high abundances, because other factors (e.g., biotic interactions) can depress abundance in otherwise suitable areas^5,6,11^. Second, density-dependent population processes can occur, thus complicating the shape of relationships.

**Figure 1:**
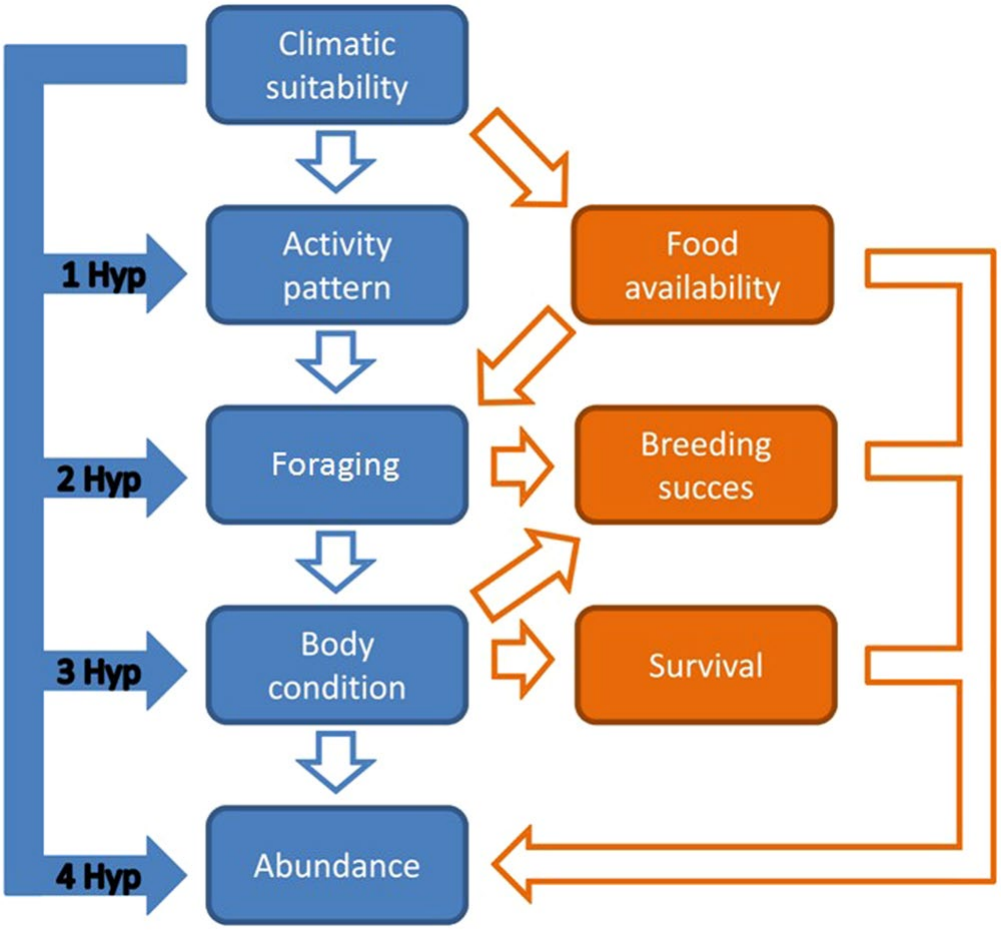
Potential relationships between climatic suitability, population parameters and species abundance. In blue the proposed pathways, in orange pathways for which no data are available in this study. Filled arrows represent hypotheses tested in the present study.

The positive relationship between environmental suitability and species abundance is probably the consequence of multiple processes acting at the population level^7^. For instance, in sites where the environment is highly favourable, individuals can spend less time in shelters and focus on activities allowing resource acquisition (e.g., foraging)^7,12–15^. In this scenario, suitable conditions can promote survival and breeding success of individuals^8,16^, leading to an increase of local abundance^7,17^ and thus to a correlation between abundance and environmental suitability^5,9,18^ (Fig. 1). Alternative pathways that can explain the relationship between environmental suitability and local species abundance involve the variation of available resources. For instance, climatic or environmental variations can influence food availability, with cascading effects on the growth rate and fitness of individuals^19–21^. Such a relationship can be particularly important for animals with highly specialized diet. More work is needed to assess the multiple and complex relationships that can occur between environmental suitability and population parameters, given that to date most of the studies have only considered the most evident correlation (i.e., suitability vs. abundance), without trying to identify the population-level processes that can determine such relationship (Fig. 1).

**Figure 2:**
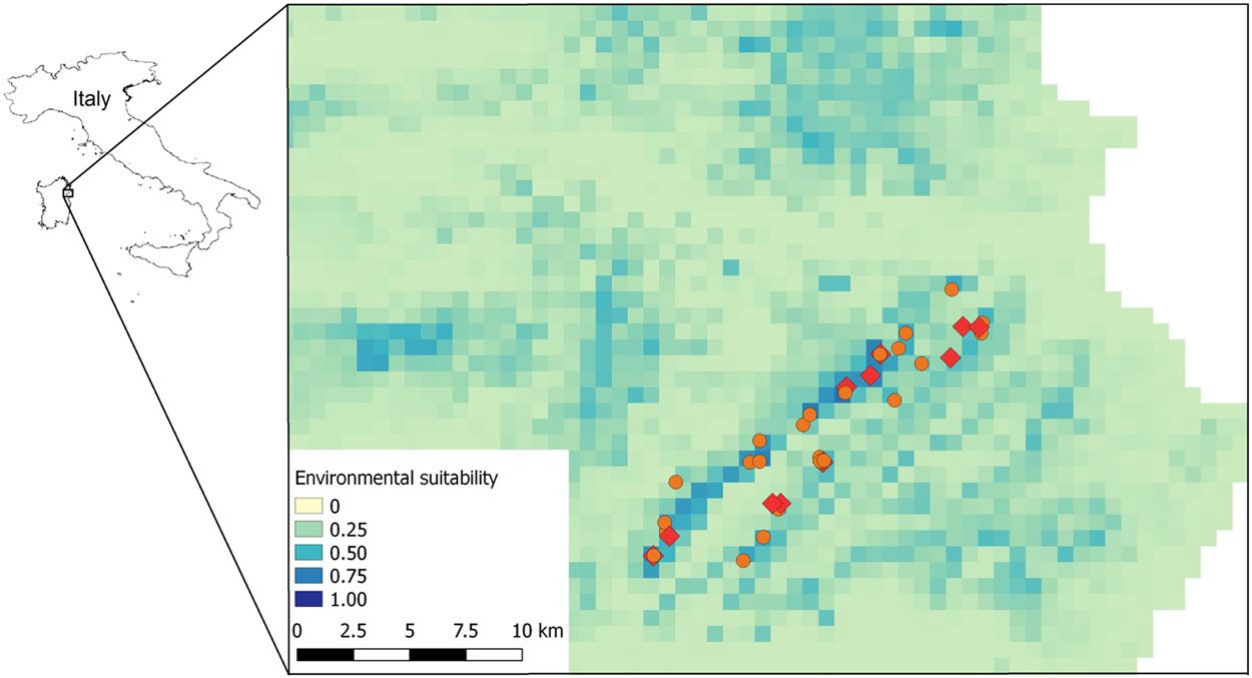
Distribution area of *Hydromantes flavus*, and environmental suitability estimated through the ensemble forecasting of multiple species distribution models. The background is limited to within 17 km north or west of the known presence records^26,60^. The map was built using the package biomod2^61^. Orange circles indicate all sites used to estimate suitability for the species; red squares represent sites surveyed in the present study.

In the present work we investigated the links between range-wide variation of environmental suitability and multiple population parameters: species abundance, activity pattern, feeding performance and body condition. We focused on the micro-endemic cave salamander, *Hydromantes flavus* (see Methods), which represents an excellent model species. *Hydromantes flavus* has a very narrow distribution (Fig. 2), facilitating sampling through the whole range. Collecting data across the entire range of a species is usually challenging^22^, but is important to accurately describe responses to environmental gradients^23^. Furthermore, *H*. *flavus* is a generalist predator of small invertebrates^24,25^, has few known predators, and there are no other terrestrial salamanders (i.e., main competitors) within its distribution range^26^, thus we do not expect major effects of biological interactions on local abundance^5^.

We predicted that spatial variation of environmental suitability can have multiple consequences on population features, and we explored multiple potential pathways (Fig. 1). *H1*) The activity hypothesis predicts that a species is more active when environmental conditions are suitable^13^. When they are active, salamanders exit their underground refuges (e.g., deep areas of caves) to reach the surface, were prey abundance is highest and most of salamander activity occurs^27–29^. If the activity hypothesis is correct we expect that, in areas with higher environmental suitability, salamanders are more often found close to the surface (i.e., they are active outside their underground refuges; see Methods). *H*2) The foraging hypothesis predicts that in highly suitable areas individuals can devote more time to forage and/or can find higher food availability, thus we expect better foraging performance. *H3*) The body condition hypothesis predicts that longer activity and better foraging allows improving body condition (i.e., more trophic muscles, more energy stored)^30^, which is a fitness-related trait^31^. *H4*) Positive relationships between environmental suitability and activity pattern, feeding and body condition are expected to improve fitness, with potential effects on abundance^9^. Therefore, we finally predict a positive relationship between the spatial variation of suitability and local density across the whole species range.

## Results

### Environmental suitability

Overall, we obtained *Hydromantes flavus* records in 25 grid cells, which represent all the known localities of the species (Fig. 2). An ensemble SDM showed an excellent performance, with an overall true skill statistic = 0.879 (sensitivity: 100%; specificity: 87.7%; standard deviation of the true skill statistic across the models: 0.159). The SDM suggested that environmental suitability (ES) increased in the highest elevation areas of the Monte Albo (North Eastern Sardinia), where mean temperature was low and annual precipitation was high (Fig. 2, Supplementary Figs S1, S2).

Model calibration was good. The coefficient of determination between the proportion of positive cases and the identity line was high (*R*^2^ = 0.56) (Fig. S3). This suggested that the model is useful both for discriminating and for ranking, even though the discrimination capacity of the model was higher than in a perfectly calibrated model^32^. The point-biserial correlation calculated on the basis of the re-calibrated POC-plot was high (*COR* = 0.52), indicating very good calibration^33^.

### Distribution of salamanders

**Figure 3:**
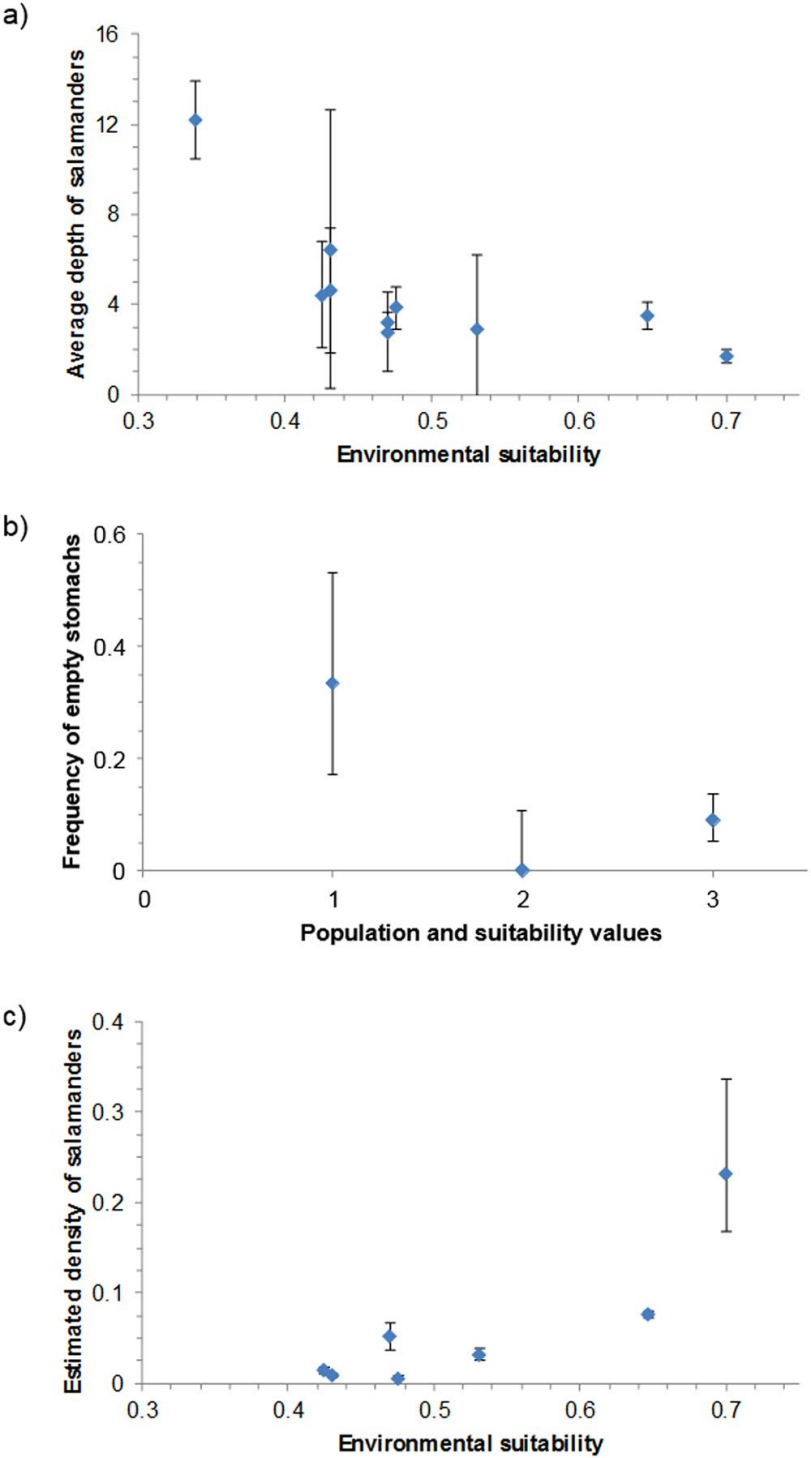
Relationship between features of salamander populations and environmental suitability. (**a**) Activity pattern (distance from cave entrance; log-transformed); (**b**) Feeding performance (frequency of empty stomachs); (**c**) Estimated density (individuals/m^2^). Bars represent standard errors.

We measured the distance from the surface (depth) of 178 salamanders from ten populations (Table 1). The average depth was highly variable among populations (Table 1), ranging from 8.5 to ~150 m. The average depth was significantly higher in sites with low ES; results were identical if we analyse data from all caves (*r* = −0.74, *N* = 10, *P* = 0.014; Fig. 3a), or if we only considered caves for which at least 5 depth measurements were available (*r* = −0.73, *N* = 8, *P* = 0.038).

**Table 1:** Monitored sites (caves).

Site	Lat	Long	Elevation	ES	Surveys	Salamander depth	Empty stomach	Residual Index	Estimated population size	Estimated density
Site 1	40.49	9.59	267	0.431	9	41.75 ± 6.14	8/24	(51) −0.140 ± 0.02	10.06 (8–13)	0.008
Site 2	40.51	9.61	116	0.470	7	7.75 ± 1.75	—	(10) −0.030 ± 0.04	—	—
Site 3	40.51	9.61	116	0.470	7	10.3 ± 0.46	—	(5) −0.149 ± 0.06	6.96 (5–9)	0.051
Site 4	40.56	9.64	777	0.701	5	3 ± 0.29	—	(3) 0.241 ± 0.06	5.50 (4–8)	0.231
Site 5	40.46	9.52	1029	0.647	10	12.36 ± 0.58	15/166	(158) −0.001 ± 0.01	103.59 (98–109)	0.077
Site 6	40.51	9.61	107	0.343	6		—	(1) −0.075	—	—
Site 7	40.47	9.53	679	0.531	5	8.5 ± 3.32	0/22	(29)−0.010 ± 0.03	9.7 (8–12)	0.031
Site 8	40.54	9.65	265	0.476	5	14.9 ± 0.94	—	—	5.69 (5–8)	0.006
Site 9	40.58	9.68	94	0.425	16	19.71 ± 2.33	—	(6) −0.027 ± 0.05	9.69 (7–13)	0.014
Site 10	40.56	9.68	116	0.420	1		—	(6) 0.040 ± 0.03	—	—
Site 11	40.57	9.64	954	0.726	1		—	(21) 0.060 ± 0.05	—	—
Site 12	40.55	9.62	902	0.786	1		—	(23) 0.180 ± 0.04	—	—
Site 13	40.58	9.69	50	0.339	1	148.27 ± 1.72	—	—	—	—
Site 14	40.49	9.58	349	0.431	1	21.43 ± 2.76	—	—	—	—

For each site we report: geographic coordinates; elevation (m a.s.l.); environmental suitability (ES); total number of performed surveys; average depth of observed salamanders (± SE); number of empty and sampled stomachs; body condition index (residual index ± SE; in parenthesis the *N* of salamanders); estimated population size (mean and 95% CI); estimated density (salamanders/m^2^).

### Stomach contents

We collected stomach contents from 212 salamanders in three populations (Table 1). One population inhabited an area with limited suitability (ES = 0.431), while the other two were found in areas highly suitable (ES > 0.53). Thirty-seven stomach contents showed only unidentifiable material and were therefore discarded from the analyses (13 for Site 1, 21 for Site 5 and 3 for Site 7). Twenty-three salamanders had empty stomach, while in 152 we recognized at least one prey item. The frequency of salamanders with empty stomach strongly differed among populations (*χ*^2^ = 14.52, df = 2, *P *< 0.001); empty stomachs were more frequent in salamanders living in less suitable sites (Fig. 3b). The frequency of empty stomachs was unrelated to the survey period (*χ*^*2*^ = 1.39, df = 1, *P* = 0.239). Bayesian credible intervals (CIs) confirmed that the salamanders of the two sites with the highest ES had a similar low frequency of empty stomachs, as their 95% CIs showed wide overlap, while the pattern was clearly different in the least suitable site (Fig. 3b).

### Body condition index (BCI)

**Figure 4:**
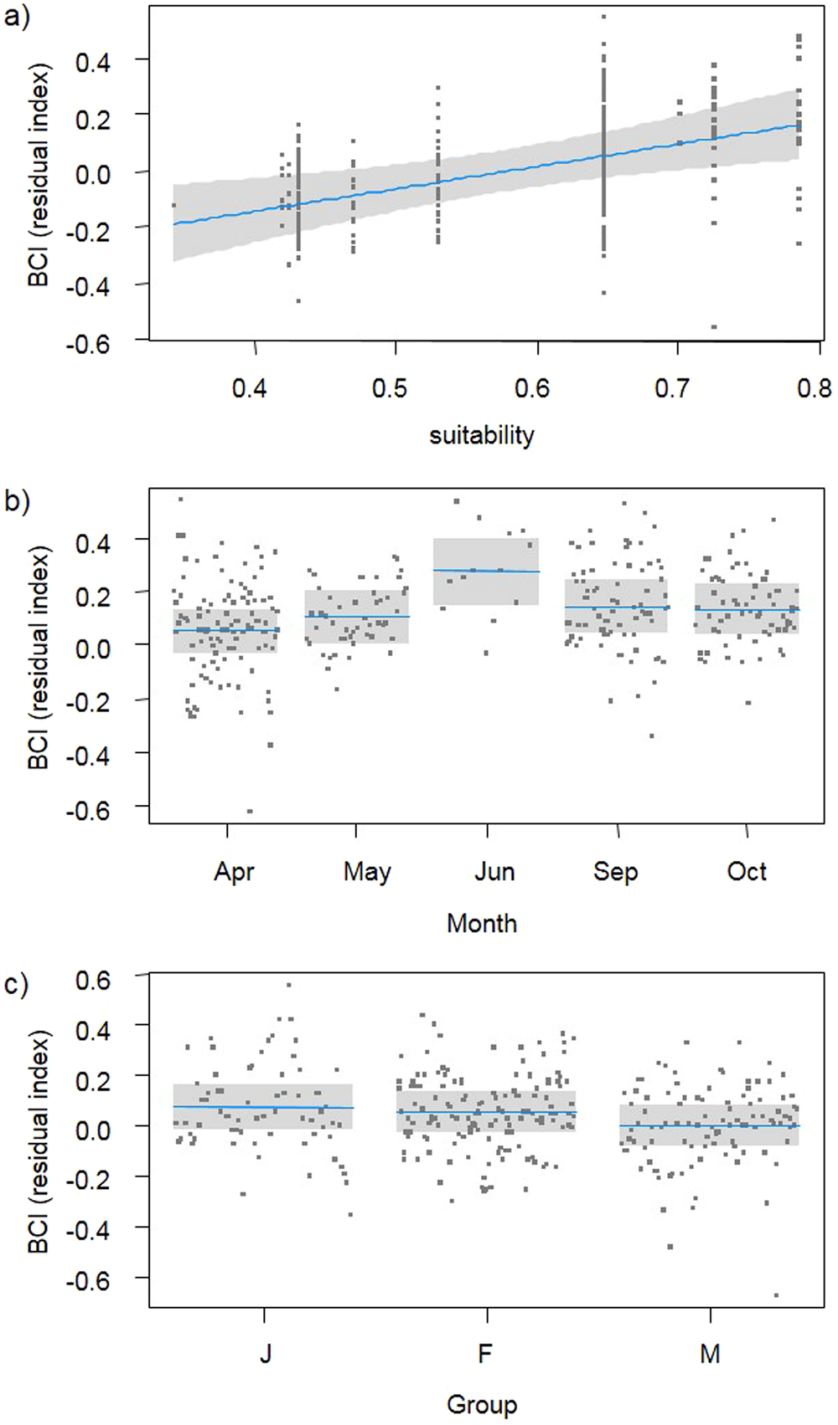
Partial regression plots of relationships between body condition index (BCI) and (**a**) environmental suitability; (**b**) month of survey; (**c**) age class and sex. Horizontal line represents mean values, while shaded box are 95% CI.

In eleven sites, we measured and weighted 313 salamanders (141 females, 104 males and 68 juveniles). The BCI did not show correlation with salamander’s length (*r* = 0.071, *N* = 313, *P* = 0.213). Average BCI was significantly higher in populations living in areas with high environmental suitability (*F*_*1*,*7*.*78*_ = 10.08, *P* = 0.013) (Fig. 4a), and showed significant variability among survey month (*F*_*4*,*285*.*17*_ = 4.73, *P* = 0.001), with better BCI in late spring-early summer (Fig. 4b). Furthermore, we detected significant variation among age/sex groups (*F*_*2*,*302*.*32*_ = 4.62, *P* = 0.01). Orthogonal contrast showed that differences between adults and juveniles were not significant (*F*_*1*,*304*.*23*_ = 3.3, *P* = 0.07) while, within adults, males showed significantly lower BCI than females (*F*_*1*,*300*.*26*_ = 6.56, *P* = 0.011) (Fig. 4c).

### Abundance and density of populations

Population abundance was estimated using a *N*-mixture model with Poisson error, as it showed lower AIC than zero-inflated models (Poisson model: AIC = 555.74, zero-inflated Poisson: AIC = 557.74). *N*-mixture models indicated a high detection probability of individuals (mean ± SE: 0.504 ± 0.029). The estimated number of salamanders was highly variable among sites, ranging from five to 103 individuals per site (Table 1); the average of density estimates across all sites was 0.06 ± 0.03 individuals/m^2^. Salamanders density strongly differed among sites, being significantly higher in areas showing the highest environmental suitability (*r* = 0.893, *N* = 7, *P* = 0.007; Table 1 and Fig. 3c).

## Discussion

Analysing multiple population parameters is essential to unravel the complex processes linking environmental suitability and individual fitness, and to understand the factors determining spatial variation of species abundance (Fig. 1). In this study, intensive sampling on multiple populations provided data on several key features of populations and allowed to test multiple hypotheses on processes that can affect the relationship between the abundance or density of a species, and environmental suitability. Even though we did not consider all the potential factors and population features (e.g., no data on breeding success or survival are available, Fig. 1), our study provides a first insight on how the interplay of multiple processes can determine the variability of abundance that can be observed across a species’ range.

Modelling the ES of *Hydromantes flavus* allowed us to identify strong heterogeneity of suitability within the Monte Albo, with suitability variation at least in part related to altitude (Figs 2 and S1, S2). At the mountain base, the microclimate is drier and warmer compared to the top, and the wet conditions at the top of the mountain probably increase suitability for *Hydromantes* (Fig. 2). *Hydromantes* are lungless salamanders that require high environmental humidity for breathing, have a narrow thermal niche, and their activity at the surface is only possible during wet, cold periods^26^. In higher ES areas, local climate shows a prolonged suitability for *Hydromantes*, a condition that likely reduces their inactivity period^27^. Indeed, when external climate is cool and moist, salamanders can exit from their refuges to prey in environments with high prey abundance^34^. As a consequence, SDM identified a suitability gradient from the lowest to the highest altitudes of the Monte Albo.

We expected a limited impact of interspecific interactions on *Hydromantes flavus* abundance^24,26^. Nevertheless, the relationship between suitability and population density was not strictly linear, as low densities were also observed in some populations living in areas with high suitability (Fig. 3c). Triangular relationships between ES and species abundance occur when biological interactions hamper populations to reach their maximum achievable abundance^5,11^. For instance, some areas of the Monte Albo are strongly affected by urbanization and mining^35^, thus human activities can determine variation of parameters not measured in this study such as vegetation features or invertebrate distribution, which can in turn affect salamander populations.

The activity hypothesis predicts that a species is more active when environmental conditions are suitable^13^. *Hydromantes* are epigean terrestrial salamanders that can spend long periods in underground environments to escape unsuitable environmental conditions, particularly during dry and hot seasons^26^. However, underground environments are not homogeneous, and are strongly influenced by external environmental conditions^36^. The activity of salamanders, and their exploitation of microhabitats, is strongly affected by a trade-off between their physiological constraints and the need of food. On the one hand, microclimatic conditions of shallow cave sectors are similar to the ones found outdoor, and these sectors can be highly unsuitable when external conditions are harsh (dry and hot)^27^. On the other hand, food availability is generally the highest close to the surface, therefore the exploitation of these sectors is important, particularly when the need of resources for growth or reproduction is high^29^. Salamander populations inhabiting the areas with most suitable climate (i.e., wet and cool climate) were more frequently found close to the surface than the ones inhabiting the least suitable sites, probably because the outdoor climatic conditions have a direct impact on the microclimate of the sites where salamanders live. Therefore, high climatic suitability favours a prolonged activity and the exploitation of cave sectors where more resources are available^29^.

The foraging hypothesis predicts better foraging performance in highly suitable sites, and the high frequency of empty stomachs that we recorded in the less suitable sites confirms that foraging events occurring in these populations may be less frequent compared to high ES sites. Sampling was performed during spring and autumn, which likely are the periods of highest activity^26^. During these seasons, salamanders probably intensify their foraging activity before aestivation and the winter diapause. Several processes can determine differences in foraging success between populations that inhabit different sites. In high ES areas salamanders can devote more time to foraging, while in low ES areas prolonged unsuitable conditions force individuals to remain in shelters^27,29^. Furthermore, it is also possible that resource availability shows spatial heterogeneity, and more resources can be available in the best sites, thus favouring preying activity^37^. Distinguishing between these non-exclusive processes is not easy; moreover, we did not measure variation of prey abundance. However, *Hydromantes* salamanders are generalist predators consuming a wide range of prey, and are able to feed on most of the underground and outdoor invertebrates^24,25^; thus we expect that prey is generally present throughout the whole species range. Nevertheless, detailed analyses are needed to assess whether the spatial variation of prey availability determines the differences in feeding performance across populations.

The body condition hypothesis predicts that longer activity period and/or better foraging allow an improvement in the conditions of individuals. Body condition of salamanders showed strong variation among populations, being significantly higher in high ES areas (Fig. 4a). The higher activity and better foraging are likely involved, determining differences in body conditions among salamanders from different areas. However, ES was not the only factor related to BCI variation. In our analyses, body condition was measured using the Residual Index. This index has an excellent performance in limiting the effect of body size on BCI^38^, thus it is not surprising that BCI differences between adults and juveniles were small. Within adults, body condition of females was significantly higher than in males (Fig. 4c), and this probably happens because females accumulate reserves for the breeding activities, which can last several months^39^. Body condition also showed a strong seasonal variation, with better body condition in June (Fig. 4b). Starting from June, outdoor conditions become extremely unsuitable for cave salamanders who move in deep underground shelters where food availability is low^27^. Therefore, *Hydromantes* likely intensively forages during the previous months in order to store more energy for aestivation^26^, and this explains the good body condition observed in June.

Positive relationships between suitability and activity pattern, feeding and/or body condition, are expected to improve fitness, with potential effects on abundance. Given that the previous hypotheses were confirmed, we expected a positive relationship between the spatial variation and local density across the whole species range. The density of salamanders showed a 30-fold variation among sites and, following our prediction, populations living in high ES areas showed the highest densities (Fig. 3c). We hypothesize that such high density is related to the co-action of multiple processes, such as prolonged activity and better feeding, which in turn improve body condition (Fig. 1). Individual body condition is a key factor which increases individual fitness and, indirectly, can affect species local abundance through different pathways^31^. First, a better body condition makes individuals able to better withstand stressing events (e.g., starvation, adverse environmental conditions), thus improving survival^40,41^. Furthermore, individuals showing better body conditions may devote more resources to breeding and parental care, improving the number and survival of the offspring^42^. These two paths are not mutually exclusive and can work synergistically. Both paths can promote population growth, especially if biological interactions do not represent a major limitation^1^. In the study species, measuring survival and breeding success is challenging, as these amphibians show an elusive breeding behaviour, and monitoring reproduction in nature is rarely possible. For instance, in 40 years of studies on *H*. *flavus*, only one egg clutch was observed during a speleological exploration^26,43^. Nevertheless, given that the detection probability of these salamanders in caves is very high, in future studies capture-mark-recapture might provide better information on the differences of survival and individual growth rate across populations^44^.

However, it should be remarked that density-dependent processes occurring at the population level are an additional issue that can complicate the relationships between suitability, local density and fitness. On the one hand, in suboptimal environments densities can be too low (e.g., hampering mating encounters), thus further limiting fitness and reducing abundance below the expected values. On the other hand, in sites with high density competition for resources increases, and this might limit food availability, with potential impacts on body conditions and fitness^45^.

Identifying the processes determining correlations between ES and local species abundance is not easy, as many factors may affect the final outcome^9^. In fact, some studies did not find positive relationships between ES and species abundance^5,7^. Integrative analyses, disentangling the multiple processes that affect population performance, are needed to unravel the complex dynamics acting at a local scale, thus helping to link local-scale population processes to those acting at broader scales, such as range-wide variation of suitability. Measuring multiple population parameters represents a key tool to understand the actual effects of environmental variation on populations. Such approach will allow to move beyond the mere measure of population abundance, to improve our understanding of the variation of fitness and population dynamics across species ranges, and also provide key data to inform explicit and mechanistic modelling of populations^3^.

## Methods

### Ethic statement

All studies were authorized by the Italian Ministry of Environment (9384/PNM of 12/05/2015 and integrations). All experiments were performed in accordance with the relevant guidelines and regulations.

### Study species and area

*Hydromantes flavus* is one of the eight European plethodontid salamanders^46^. It has a small distribution range (<90 km^2^) and is endemic to the Monte Albo in north-eastern Sardinia^26^, Italy (Fig. 2). Plethodontid salamanders are lungless and breathe mostly through the skin^26^, thus they have a narrow ecophysiological niche, requiring high moisture and relatively cold temperature^26,47^. Such microclimatic requirements are generally found in underground environments (e.g., caves), where the species can be observed throughout the year^27^, especially when outdoor conditions become unsuitable^48,49^. However, *H*. *flavus* is not an obligate cave dweller: during suitable seasons it is active outdoor, preying on invertebrates^34,50^. When underground, these salamanders usually occupy sectors not far from the surface, to be closer to food resources^29^. Underground shallow areas are strongly influenced by external climatic conditions, thus external climate influence salamanders even when they are underground^27^.

### Suitability modelling

We used correlative species distribution models to assess relationships between salamander distribution and major bioclimatic variables, and to obtain measures of broad-scale ES. We considered four bioclimatic variables: annual mean temperature, temperature seasonality, annual precipitation and precipitation seasonality (period: 1979–2013); variables were extracted from the Chelsa-climate dataset at the 30 arc-seconds resolution (approx. 920 × 700 m within the study area), which provides improved climatic estimates in landscapes with complex topography^51^. These variables represent average conditions and their variability across the year, and are major determinants of vertebrate distribution^52,53^. Furthermore, these variables are enough to explain most of the climatic variation^54^, and other important variables (e.g., winter and summer temperatures) are strongly related to linear combinations of the four variables considered. We did not include variables representing the biotic habitat^9^, because these salamanders are mostly related to fine-scale microhabitat variables that are not captured by remote sensing or broad-scale habitat maps^29^. The correlation between variables was weak to moderate (for all pairwise correlations, |*r*| <0.77). To calibrate models, we used all published presence records of the species (reviewed in^26,55^), updated with records from our own surveys (Fig. 2). To limit the effect of spatial heterogeneity in sampling efforts, we considered only one presence record per grid cell. SDM were built using five modelling approaches: Generalized Additive Models, Boosting Regression Trees, Classification Trees, Multiple Adaptive Regression Splines and Random Forests. Models were calibrated using a 67% random sample of the presence data and evaluated against the remaining 33% data using the True Skill Statistic (TSS)^56^. To improve reliability of models, we restricted the geographical background to a small region potentially accessible to the species^57–59^. Background data were limited to within 17 km from the known presence records (Fig. 2), which is the maximum distance between *H*. *flavus* populations belonging to the same genetic cluster^60^. This analysis was repeated five times, thus providing a fivefold cross-validation; models were run using the package biomod2 (version 3.3–7)^61^ in R 3.3.3^62^. Given that alternative SDM can provide variable outputs, ensemble forecasting of the different SDM^63^ was then used to obtain an overall suitability map. Suitability was calculated as the sum of occurrence-probability projections made by the five modelling techniques run over the three subsamples, weighed by their TSS^64^. It should be remarked that TSS is prevalence independent when making binary predictions, but not when making continuous predictions^65^. We used two approaches to assess the calibration of the model. First, we built calibration plots and calculated the coefficient of determination between the proportion of positive cases and the identity line^32^. Second, as our SDMs were based on presence-only data, we also used POC-plots to build presence-only calibration plots^33^. Calibration was calculated using point-biserial correlation, on the basis of the re-calibrated POC-plot^33^.

### Surveys

From June 2013 to May 2017, we performed multiple detailed surveys in a total of 14 underground sites (caves) where *Hydromantes flavus* was known to be present, covering the whole range of the species^26^ (Table 1, Fig. 2). Surveys were performed from April to October, the period in which *Hydromantes* salamanders are more active and show the highest abundance and detectability in underground environments^27,66^. During the first survey, we divided each cave in 3-m longitudinal sectors, and measured the maximum height of the ceiling and the maximum width of each sector^49^. These measures were then used to estimate the explored cave surface. Subsequently, we measured multiple features of the populations: distribution of salamanders within these caves, stomach content, body condition and density. Some of these population features required sampling during specific months and thus, due to sampling constraints (i.e., restricted sampling periods, need of speleological equipment), it was impossible to measure all the population features for all the caves, nevertheless for all the features we gathered data covering the whole range of the species (Table 1).

### Distribution of salamanders in caves

To measure salamander distribution we performed surveys in May-June, as in this period the exploitation of cave environments is the highest^27^. Since several caves received multiple surveys (11 caves surveyed; average: 4.82 surveys per cave; Table 1), for each cave we considered the survey with the highest number of observations. We measured the depth (distance from the cave entrance) of all salamanders detected inside the caves, using a 30-m fibreglass tape meter and a laser-meter (accuracy 2 mm). We used the correlation between the average depth of salamanders (log-transformed) and environmental suitability to test the activity hypothesis. Estimates of average depth might be less robust in caves for which we captured only a few individuals, therefore we repeated such analysis considering only caves with ≥5 individuals detected in at least one survey; results were identical in both cases. Dataset is shown in Supplementary Table S1.

### Stomach content

In three populations (Table 1), we performed stomach flushing, which is an unharming technique widely used to check stomach contents of amphibians^67^. For each population, we performed two capture sessions in spring and two in autumn (period: autumn 2015-spring 2017; total: four samplings per site). Stomach flushing was performed using a 5 ml syringe connected to a 1 mm ∅ plastic pipe; the end of the pipe was introduced in the oral cavity of the salamander and 5 ml of water was gently injected in the stomach. Reflux was canalized by a funnel into a plastic jar. The obtained stomach contents were preserved in 75% ethanol and then identified using an optical microscope. Stomach contents were separated in two groups: empty (no items detected) and full (at least one food item was observed)^50^.

To assess if the frequency of empty stomachs differs among caves (i.e., to test the foraging hypothesis), we run the binomial Generalized Linear Models (GLM) using the stomach condition (empty/full) as dependent variable, while season (autumn/spring) and cave identity were used as independent variables. We also used the Bayesian equal-tailed Jeffreys intervals (package MKmisc;^68^) to estimate 95% CI of the frequency of empty stomachs across populations, as Jeffreys intervals are a robust approach for the estimation of binomial CI^69^. The dataset is published in^50^.

### Body condition index

Captured salamanders were weighted (using an electronic scale; precision: 0.01 g) and measured (total length; using a plastic ruler). To include the largest number of sites in this analysis, we also considered a cave where only one salamander was measured (Site 6, Table 1); results did not change if this cave was excluded. For each individual we calculated the Residual Index, which is the difference between the observed and the predicted body mass of animals and is considered among the most reliable body condition indexes^38,70^. To calculate the residual index, we regressed weight against total length of salamanders, and for each individual we extracted the residuals of the regression^38,70^. Weight and length were log-transformed to improve linearity. We considered the total length because these animals often store fat also in the tail^71^. We identified age classes and sex on the basis of secondary sexual characters and body size. Salamanders with male sexual characters (mental glands and premaxillary teeth) were considered adult males; individuals without male characters but ≥80 mm were considered adult females (80 mm is the size of the smallest observed adult males); individuals <80 mm were considered juveniles^26^.

Populations were surveyed in different periods of the year (from October 2015 to April 2016), and salamander body features were measured in 20 surveys performed in 11 populations (average: 1.8 surveys per site). To avoid pseudoreplication, for each population we only considered one survey per month, selecting the one with the highest number of measured salamanders, as individual identification was impossible in most of cases^72^. Body weight can show seasonal variation. To test the body condition hypothesis, we ran a linear mixed model (package lme4;^73^) considering BCI of salamanders as dependent variable, ES, month of survey and group (m/f/j) as independent variables, and population identity as random factor. Sample size was not homogeneous among populations, thus residual degrees of freedom were approximated following Satterthwaite^74^. Given that we detected significant BCI differences between the tree groups, we used orthogonal contrast to test whether there are differences between juvenile and adults and, within adults, between males and females. Dataset is shown in Supplementary Table S2.

### Population abundance and density

In seven caves we performed repeated visual encounter surveys in a short period, to estimate salamander abundance. During each survey, the same person dedicated 7.5 min. of observation to each 3-m long longitudinal sectors counting the active salamanders^66^. Each cave was surveyed five times in 2016, from May 4^th^ to June 27^th^. In this period, cave occupancy and detection probability of salamanders is the highest, immigration/emigration to/from the cave is minimum, and no hatches are known to occur^27,43^, thus allowing to meet the closed population assumptions of *N*-mixture models^75^.

We used *N*-mixture models to estimate population size on the basis of repeated counts^76^. This approach provides accurate estimates of actual population size, particularly in species with high detection probability such as cave salamanders^77^. We used Akaike’s Information Criterion (AIC) to select the most appropriate error distribution (Poisson or zero-inflated Poisson); negative binomial models were not considered as they can produce infinite abundance estimates^78^. We used Empirical Bayes methods to estimate the posterior distribution of the abundance (mean and 95% Bayesian credible intervals, CI)^79^. Surveyed surface was strongly variable among caves (33–1229.61 m^2^), therefore we calculated salamander density to allow comparison among sites. We calculated population density on the basis of abundance estimates and the surveyed surface of caves, and then assessed the correlation between population density (square-root transformed) and ES. *N*-mixture models were run using the unmarked package in R^80^. Dataset is shown in Supplementary Table S3.

Spatial autocorrelation can influence the outcome of statistical tests, therefore we used Moran’s *I* to assess the occurrence of residual autocorrelation in all regression and correlation analyses^81^. For all regression models, residual autocorrelation was weak and not significant (in all tests, Moran’s *I* ≤ 0.2, all *P* > 0.05), suggesting that spatial autocorrelation was not an issue to our results. For SDMs, spatial autocorrelation of residuals was significant but weak (Moran’s *I* = 0.12, *P* < 0.01).

## Electronic supplementary material

Supplementary information

Dataset 1

Dataset 2

Dataset 3
