# Supplementary material for: Environmental suitability models predict population density, performance and body condition for microendemic salamanders
Source: Sci Rep. 2018 May 14;8:7527. doi: 10.1038/s41598-018-25704-1 (PMC5951833; doi:10.1038/s41598-018-25704-1)

Environmental suitability models predict population density, performance and body condition for microendemic salamanders

Enrico Lunghi, Raoul Manenti, Manuela Mulargia, Michael Veith, Claudia Corti, Gentile Francesco Ficetola

Supplementary Material

Figure S1. Altitudinal map of the study area. Orange circles indicate all sites used in SDM analysis to estimate ES for the species; red squares represent surveyed site in the present study (see Fig. 2). The map was performed using the program QGIS (http://www.qgis.org); source of raster data http://chelsa-climate.org/.

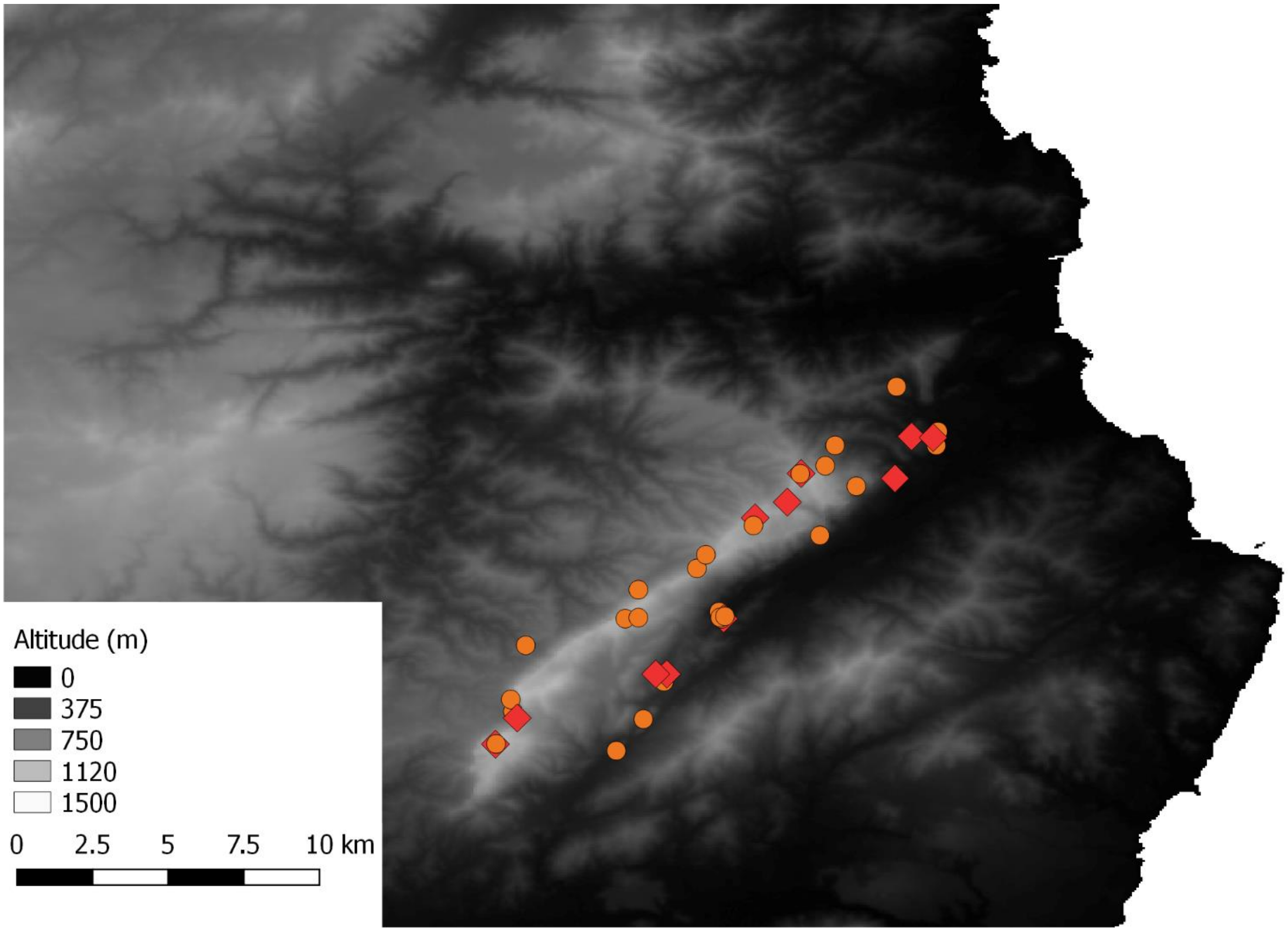

Figure S2. Relationship between altitude and climatic variables within the study area.

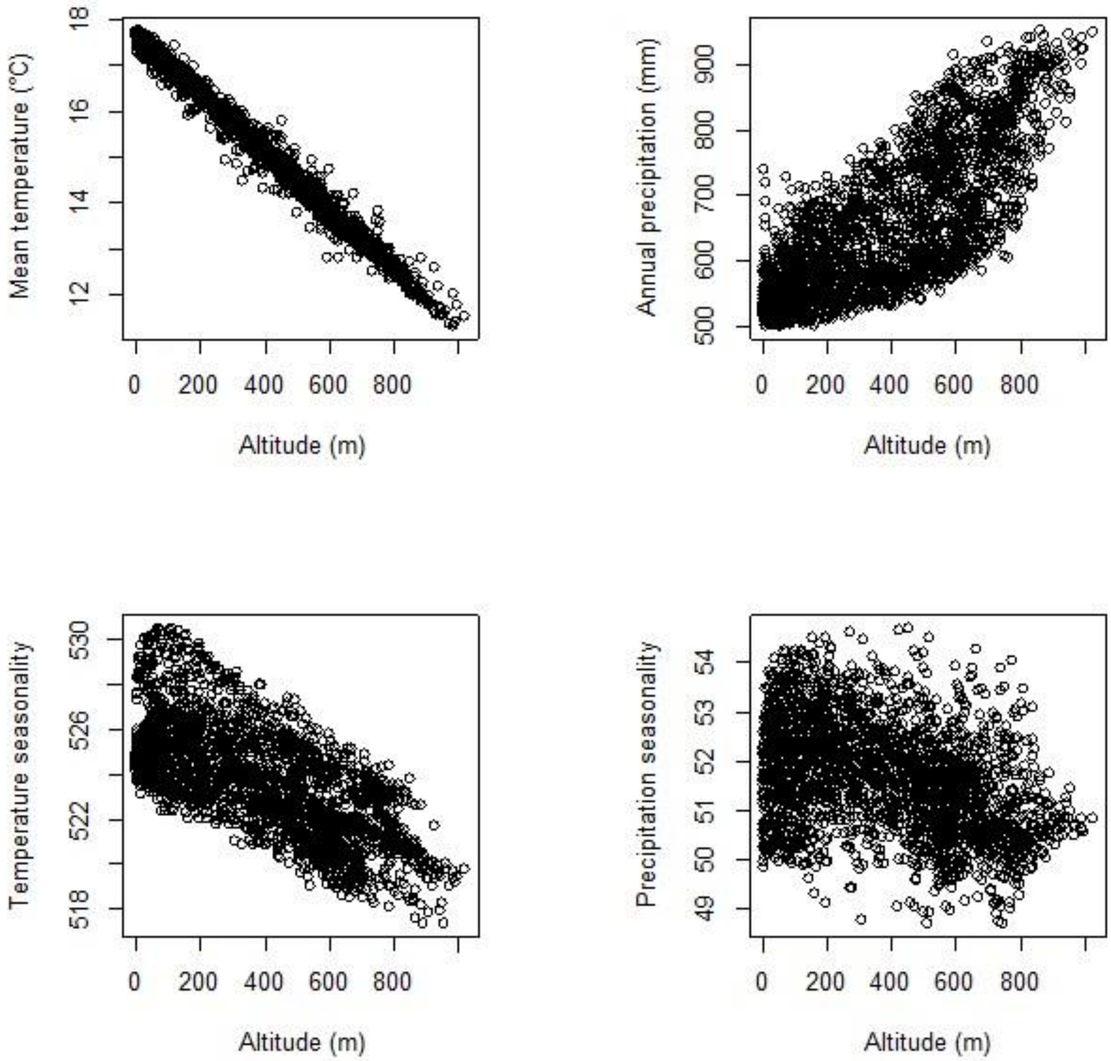

Figure S3. Calibration plot, showing the suitability estimated by the model against the observed proportion of positive cases (y-axis) for equal-size probability intervals (bins). Black dots represent the observed proportion of positive cases at each bin; the orange line is the identity line indicating perfect calibration (intercept = 0 and slope = 1).

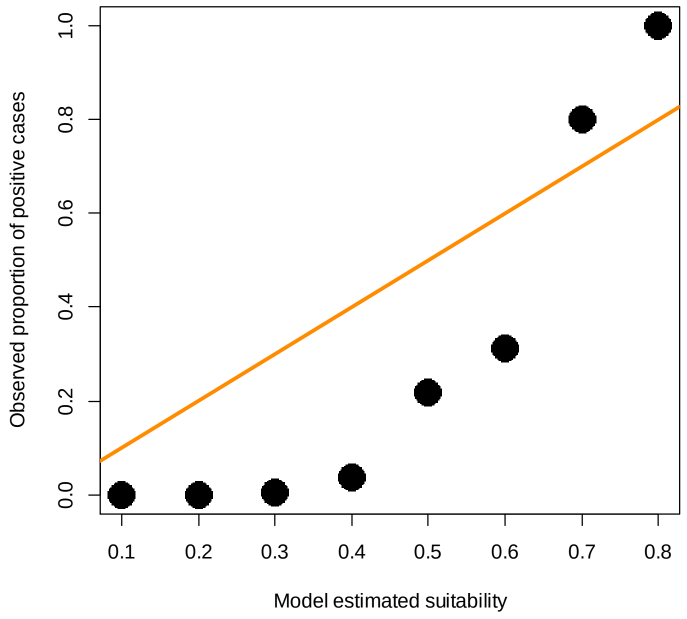

Supplement: Supplementary file 1 — Supplementary information [file 41598_2018_25704_MOESM1_ESM.pdf]
